# Pattern of Antibiotic Prescriptions in Chinese Children, A Cross-Sectional Survey From 17 Hospitals Located Across 10 Provinces of China

**DOI:** 10.3389/fped.2022.857945

**Published:** 2022-07-14

**Authors:** Jiaosheng Zhang, Xiang Ma, Lanfang Tang, Daiyin Tian, Li Lin, Yanqi Li, Gen Lu, Ligang Si, Wenshuang Zhang, Jing Qian, Lijuan Wu, Gang Liu, Wei Li, Qing Cao, Keye Wu, Yuejie Zheng, Jikui Deng, Yonghong Yang

**Affiliations:** ^1^Department of Infectious Diseases, Shenzhen Children's Hospital, Shenzhen, China; ^2^Department of Respiratory, Jinan Children's Hospital and Children's Hospital Affiliated to Shandong University, Jinan, China; ^3^Department of Respiratory, Children's Hospital Zhejiang University School of Medicine, Hangzhou, China; ^4^Department of Respiratory, Children's Hospital of Chongqing Medical University, Chongqing, China; ^5^Department of Respiratory, The Second Affiliated Hospital and Yuying Children's Hospital of Wenzhou Medical University, Wenzhou, China; ^6^Department of Respiratory, Xi'an Children's Hospital, Xi'An, China; ^7^Department of Respiratory, Guangzhou Women and Children's Medical Center, Guangzhou, China; ^8^Department of Respiratory, The First Hospital of Haerbin Medical University, Harbin, China; ^9^Department of Respiratory, Tianjin Children's Hospital, Tianjin, China; ^10^Department of Respiratory, Children's Hospital Attached to The Capital Institute of Pediatrics, Beijing, China; ^11^Clinical Laboratory, Bao'an Maternity and Child Health Hospital, Shenzhen, China; ^12^Department of Infectious Diseases, Beijing Children's Hospital Affiliated to Capital Medical University, Beijing, China; ^13^Department of Pediatric Respiratory, The First Hospital of Jilin University, Changchun, China; ^14^Department of Infectious Diseases, Shanghai Children's Medical Center, Shanghai Jiaotong University School of Medicine, Shanghai, China; ^15^Department of Cardiothoracic Surgery, Shenzhen Children's Hospital, Shenzhan, China; ^16^Department of Respiratory, Shenzhen Children's Hospital, Shenzhen, China; ^17^Beijing Pediatric Research Institute, Beijing Children's Hospital Affiliated to Capital Medical University, National Center for Children's Health, Department of Internal Medicine, Shenzhen Children's Hospital, Beijing, China

**Keywords:** antibiotic, inpatients, pattern, children, China, AWaRe classification

## Abstract

**Objectives:**

Use of Broad-spectrum antibiotics is related closely to increasing antimicrobial resistance. Reports on antibiotic prescriptions for Chinese children were rare. We described the prescribing patterns of antibiotic prescriptions for Chinese children from 2017 to 2019 based on the Anatomical Therapeutic Chemical Classification (ATC classification); the Access, Watch, and Reserve (AWaRe) classification from the World Health Organization (WHO), and the Management of Antibiotic Classification in China.

**Methods:**

A 1-day point-prevalence survey (PPSs) on antibiotics prescribing for Chinese children was conducted in hospitalized children from 17 centers in 10 Chinese provinces from 1 September 2017 to 30 November 2019.

**Results:**

A total of 4,982 antibiotic prescriptions for Chinese children were included in the analysis. There were 76 types of antibiotic agents in total, 22 (28.9%) of which accounted for 90% of all antibiotic prescriptions. The top-three antibiotics prescribed for children were azithromycin (684, 13.7%), ceftriaxone (508, 10.2%) and latamoxef (403, 8.1%). Third-generation cephalosporins (1,913, 38.4%) were the most commonly prescribed antibiotic classes. On the basis of the AWaRe classification, the Watch group antibiotics accounted for 76.3% and Access group antibiotics accounted for 12.1% of all antibiotic prescriptions. On the basis of the China classification, we showed that 26.5% of antibiotic prescriptions were in the Unrestricted group, 53.6% in the Restricted group, and 14.5% in the Special group.

**Conclusion:**

The proportion of antibiotics included in the Watch group and the Special group was high in children in China. The AWaRe classification and China classification for antibiotic prescriptions could be used to supply detailed data for antibiotic stewardship as a simple metric.

## Highlights

- There Was a Large Amount of Data on Antibiotic Usage in Chinese Children.
- This Study Focused on the Pattern of Children not Including Neonates.
- Detailed Antibiotic Classification Information of Every Group Was Described, It Will be Useful to Develop Antimicrobial Management Measures With Specific Details.
- China Classification and WHO AWaRe Classification Were Both Used to Describe the Pattern of Antimicrobial Prescriptions, Which Could be Understood More Clearly.

## Introduction

Antimicrobial resistance has become a global threat to public health in the 21^st^ century. The World Health Organization launched a global action plan for antimicrobial resistance. One of the key strategies is to carry out surveys on antibiotic prescribing to optimize use of antimicrobial agents.

Exposure to antibiotics is a major driver of antimicrobial resistance. Rational use of antibiotics is a major tool to slow the emergence of resistant pathogens, especially multidrug-resistant organisms. Broad-spectrum antibiotics have higher resistance potential. It is particularly important to assess the relative use of broad-spectrum antibiotics for children in China.

Data reports on the pattern of antibiotics prescribing for Chinese children are rare. Some sporadic studies have reported the use of antibiotic agents for a certain type of infection (e.g., acute upper respiratory tract infection) but such results cannot represent the overall picture of antibiotics prescribing for Chinese children. Developing a series of strategies for optimizing antimicrobial use based on such results is difficult. A lack of practical and simple tools to assess the relative proportion of broad-spectrum antibiotics, the relevant data are limited.

In 2017, the 21^st^ WHO Expert Committee divided antibacterial agents for children into three categories: Access, Watch, and Reserve (AWaRe). These categories were modified in 2019 (1). The Access group of antibiotics has relatively low potential for resistance and antibiotics in this group are recommended as first-line treatment for the main infectious diseases in children. The Watch group includes antibiotics that have higher resistance potential and need to be monitored as priority agents. The Reserve group of antibiotics should be treated as the “last-resort” for patients with infections caused by multidrug-resistant bacteria.

The antibacterial drugs were divided into three subgroups: Unrestricted, Restricted, and Special group based on Chinese antibiotic classification (2). The Unrestricted group includes antibiotics that are safe, affordable, and effective, with little impact upon bacterial resistance. The Restricted group includes antibiotics that have a high potential for bacterial resistance and/or a high cost. The Special use group includes antibiotics that can induce serious adverse effects, are expensive, and/or have a high probability of inducing bacterial resistance.

There were some obvious differences between the AWaRe classification and the Management of Antibiotic Classification in China. For example, second-generation cephalosporins and carbapenems are classified in the Watch group in the AWaRe classification. Compared with second-generation cephalosporins, carbapenems are very different and should be prescribed for children with infections caused by multidrug-resistant pathogens, and are more expensive. Based on antibiotic classification in China, carbapenems are separate from second-generation cephalosporins and third-generation cephalosporins, and classified into a special group of antibiotics. A combination of the China Classification and WHO AWaRe could provide a clearer picture of antibiotic prescribing for children in China.

One multicenter point prevalence survey showed the pattern of antibiotic prescriptions for hospitalized children in China based on the WHO AWaRe classification. However, the data were not separate from overall data (which included children and neonates), whereas the antibiotics prescribed for children and neonates were significantly different (3). Moreover, the pattern of antibiotic prescribing based on the classification in China that applied to daily management in China, and detailed information on antibiotic agents in each group was lacking.

Here, we aimed to present the general prescription patterns for antibiotics given to children. We focused on the proportion of broad-spectrum antibacterial agents for common indications in children for 3 years from 2017 to 2019 based on the Anatomical Therapeutical Chemical (ATC) Classification, the WHO AWaRe classification, and the Management of Antibiotic Classification in China.

## Methods

### Data Collection

A 1-day point-prevalence survey was completed in September–November annually from 2017 to 2019. 17 participating centers from 10 provinces in China joined in for this project. Children (age range: 29 days to 18 years old) who were hospitalized in participating hospitals were included in this survey. The wards involved were as follows: internal medicine wards (respiratory, infectious diseases, general pediatric wards, and neonatology wards), pediatric surgery, and intensive care units (Neonatal ICUs and Pediatric ICUs) from 2017 to 2019. This survey collected detailed information about patients who were prescribed antibiotics, such as their ages, body weights, and the antimicrobial agents that they used (as well as their doses and routes of administration). The total number of patients in the wards in each hospital was also documented. The indications for antibiotics were as follows: pneumonia; bronchitis; viral co-infection with bacteria treatment for surgical diseases; surgical prophylaxis; medical prophylaxis; sepsis; catheter-related bloodstream infection; central nervous system (CNS) infections; skin/soft tissue infections (SSTI); urinary tract infections (UTI); joint/bone infections; cardiac infections; acute otitis media (AOM); upper respiratory tract infections (URTI); lymphadenitis; gastrointestinal (GI) tract infections; pyrexia of unknown origin (PUO); febrile neutropenia/fever; other; and unknown. The reasons for antibiotic use was selected from the structured entry diagnosis. If the reason was not in the catalog, “other” was selected. If the reason was not clear, “unknown” was selected. The indications for which antibiotic prescriptions were <10 would be merged. We examined the patterns of antimicrobial use of the pediatric units in joined hospitals. 11 of the centers were children's hospitals, three were general hospitals, and three were healthcare centers for women and children. All hospitals were tertiary and teaching hospitals. The ethics committees at Shenzhen Children's Hospital and all participating hospitals approved the procedures in this study.

An internet-based Electronic Data Capture (https://garpec-31.mobilemd.cn/login.aspx) was used to collect results. All participating centers logged into this database using a specific username and password and uploaded their data online. The data from 2017 to 2019 were merged for analysis.

Antibiotics for systemic use (intravenous, intramuscular, and oral antibiotics) were analyzed. Only antibacterial agents were analyzed, whereas antifungal, antiviral, antitubercular agents, and antiparasitic drugs were excluded. Children with infections were classified as having community-acquired infections (CAIs) if the onset of infection was ≤ 48 h after admission, and hospital-acquired infections (HAIs), if the onset was >48 h after admission.

According to Chinese antibiotic classification, the antibacterial drugs were classified into three groups: Unrestricted, Restricted, and Special group. There was no correspondence in the lists of the three groups between the Chinese and WHO classifications. The Unrestricted group includes antibiotics like ampicillin, azithromycin for oral, and cefaclor, which are safe, affordable, and effective. The Restricted group includes antibiotics like cefoperazone/sulbactam, which have a higher potential bacterial resistance and/or a higher price. The Special use group includes antibiotics like vancomycin and meropenem, which can induce serious adverse effects and are expensive. The Special antibiotic group lists were the same in different districts and hospitals across China. The Unrestricted and Restricted antibiotic groups were slightly different in different provinces and hospitals. In this study, the lists of Unrestricted and Restricted antibiotic groups were integrated by merging the lists applied in the Tianjin Children's Hospital (representing hospitals in the North) and Shenzhen Children's Hospital (representing hospitals in the South).

The antibiotic list of WHO AWaRe groups, as well as Unrestricted, Restricted, and Special groups of antibiotics in China, is shown in Supplementary Table 1.

Antibiotics that were not on the list of the WHO ATC classification were classified into a similar group according to their pharmacological characteristics. For example, cefathiamidine was classified as a first-generation cephalosporins.

### Statistical Analysis

Patterns of antibiotic prescription were described with a drug utilization (DU) of 90% (defined as the number of antibiotics that accounted for 90% of the total antibiotics prescriptions). Antibiotic prescriptions in this multi-center point prevalence survey were classified on the basis of the ATC classification, AWaRe classification, and antibiotic classification in China.

For statistical analyses, Microsoft Excel 2007 (Microsoft, Redmond WA, USA) and SPSS 22.0 (IBM, Armonk, NY, United States) were used.

## Results

Seventeen centers located in 10 Chinese provinces participated in this survey. Antibiotic prescribing data from 5,996 hospitalized children were collected and 4,043 (67.4%) patients were prescribed antibiotics for treatment or prophylaxis. The sum of antibiotic prescriptions was 4,982. A total of 3,154 (78.0%) children were prescribed only one antibiotic and 889 (21.98%) children received combination antibiotic therapies. There were 614 (614/4,982, 12.3%) prescriptions for targeted use and 4,368 (4,368/4,982, 87.7%) for empirical use. Of 4,982 antibiotic prescriptions, 79 (1.7%) antibiotic prescriptions were for hospital-acquired infections, 4,335 (93.4%) for community-acquired infections, and 230 (4.9%) were for an unknown cause. Meanwhile, 331 (6.6%) prescriptions were used for antibiotic prophylaxis and 4,651 (93.4%) for treatment. Among the antibiotics for hospitalized children in China, 4,676 (93.9%) were given *via* the intravenous route and 306 (6.1%) were administered *via* the oral route. The rate of antibiotic prescribing in pediatric wards in general hospitals (71.5%) was the highest, followed by in children's hospitals (68.9%) and maternal and children's hospitals (49.9%) (*P* < 0.05). The antibiotic prescribing rate in provincial hospitals (71.1%) was higher than in district hospitals (52.8%) (*P* < 0.05). The characteristics of antibiotic prescriptions in different hospitals are shown in Table 1.

**Table 1:** The characteristics of participating hospitals by frequency of 4,982 antibiotic prescriptions from 2017 to 2019.

**Hospital code**	**Level**	**Type**	**Province**	**District**	**Ward types**	**Total patients**	**Patient prescribed antibiotics**	**Median age(month)**	**Rates of antibiotic therapy (%)**	**No. of antibiotic prescriptions**	**No. of antibiotic prescriptions per patient**
H 1	Tertiary	Children's	Shandong	Provincial	M, S, I	1128	864	19	76.6	1,089	1.3
H 2	Tertiary	Children's	Zhejiang	Provincial	M, S, I	909	611	15	67.2	731	1.2
H 3	Tertiary	Children's	Chongqing	Provincial	M, S, I	865	511	15	59.1	670	1.3
H 4	Tertiary	Children's	Zhejiang	Municipal	M, S, I	631	368	36	58.3	424	1.2
H 5	Tertiary	Children's	Shaanxi	Provincial	M,S,I	391	306	24	78.3	386	1.3
H 6	Tertiary	Children's	Tianjin	Provincial	M, I	236	218	48	92.4	273	1.3
H 7	Tertiary	Children's	Guangdong	Municipal	M, S, I	345	188	24	54.5	217	1.2
H 8	Tertiary	General	Heilongjiang	Provincial	M, S, I	255	172	36	67.5	191	1.1
H 9	Tertiary	Maternal and children's	Guangdong	Provincial	M, S	274	163	16.5	59.5	190	1.2
H 10	Tertiary	Children's	Beijing	Provincial	M, S, I	227	161	48	70.9	181	1.1
H 11	Tertiary	General	Jilin	Provincial	M, S, I	156	125	36	80.1	169	1.4
H 12	Tertiary	Children's	Beijing	National	M	161	123	9	76.4	160	1.3
H 13	Tertiary	Children's	Shanghai	National	M, S, I	141	110	16	78.0	147	1.3
H 14	Tertiary	Maternal and children's	Guangdong	County	M, S, I	217	74	24	34.1	95	1.3
H 15	Tertiary	Children's	Shanghai	Provincial	M	30	27	24	90.0	32	1.2
H 16	Tertiary	Maternal and children's	Guangdong	Provincial	I	20	18	4	90.0	23	1.3
H 17	Tertiary	General	Shandong	Provincial	I	10	4	1	40.0	4	1.0
Total						5,996	4,043		67.4	4,982	1.2

*M, medical wards; S, surgical wards, I, intensive care units*.

### Indications for Antibiotics in Children in China From 2017 to 2019

The most three common reasons for antibiotic use in children in our survey were pneumonia (2,632, 52.8%), treatment for surgical diseasees (633, 12.7%), and bronchitis (362, 7.3%). The detailed distribution of indications for Chinese children is shown in Table 2.

**Table 2:** Indications of 4,982 antibiotic prescriptions for Chinese children.

**Indications for antimicrobial prescribing**	**Frequency**	**Percentage (%)**
Pneumonia	2,632	52.8
Treatment for surgical disease	633	12.7
Bronchitis	362	7.3
Central nervous system infections	253	5.1
Surgical prophylaxis	232	4.7
Sepsis	192	3.9
Upper respiratory infections	113	2.3
Gastrointestinal tract infections	103	2.1
Medical prophylaxis	99	2.0
Other	77	1.5
Viral co-infection with bacteria	76	1.5
Pyrexia of unknown origin (PUO)	53	1.1
Skin/soft-tissue infections	48	1.0
Pertussis	34	0.7
Urinary tract infections (UTI)	34	0.7
Lymphadenitis	17	0.3
Unknown	14	0.3
Pyogenic tonsillitis	10	0.2
Total	4,982	100

### Prescription Pattern Based on the Antibiotic

Of the 4,982 antibiotic prescriptions for children, there were 76 types of antibiotic agents in total, and 22 (28.9%) antibiotic agents accounted for 90% of antibiotic prescriptions.

**Figure 1:**
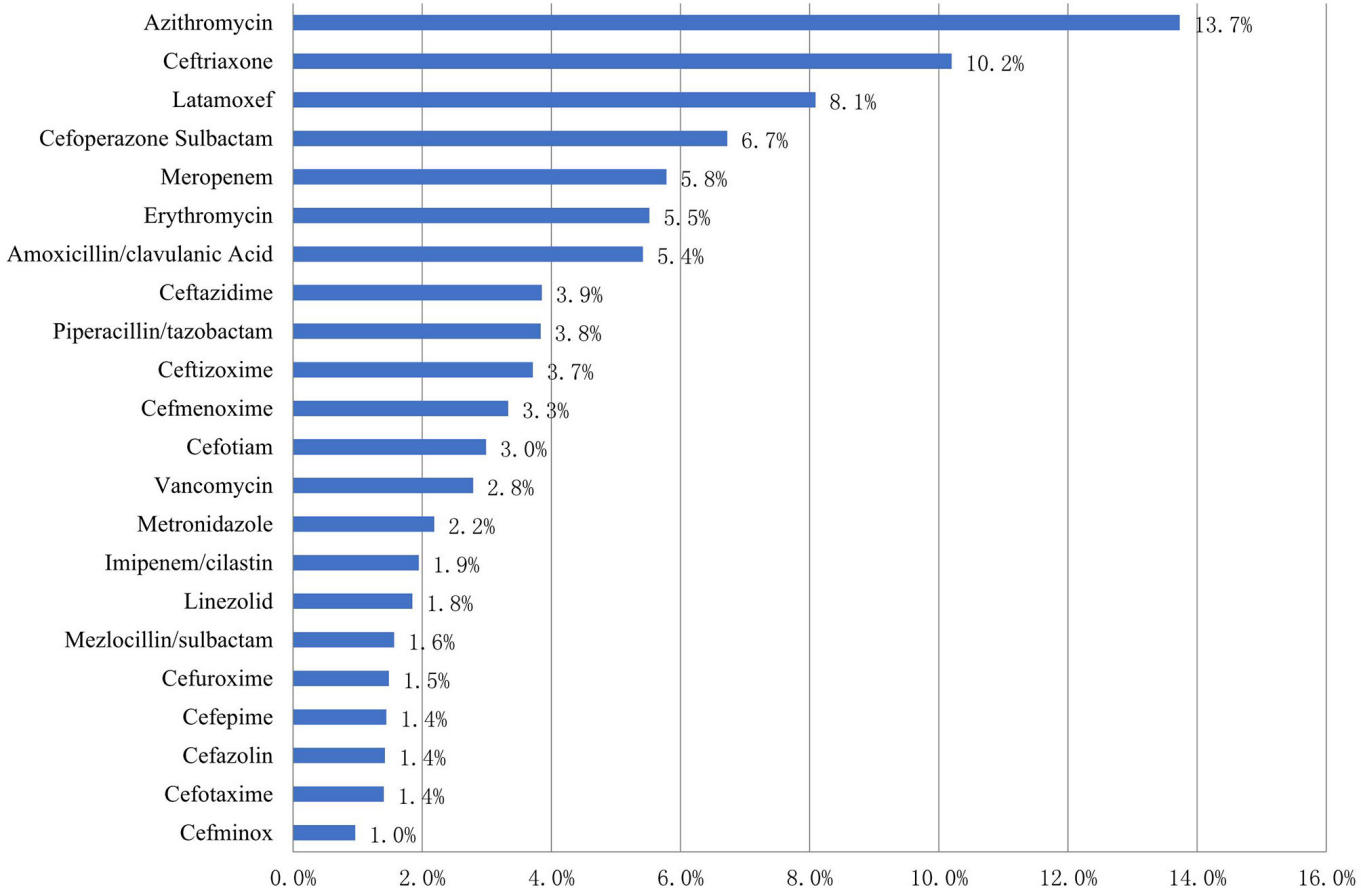
Patterns of antibiotic prescribing to Chinese children by drug utilization 90% in 2017–2019 (%).

The five most common antibiotics prescribed for children (which accounted for 44.5% of all antibiotic use) were azithromycin (684, 13.7%), ceftriaxone (508, 10.2%), latamoxef (403, 8.1%), cefoperazone sulbactam (335, 6.7%), and meropenem (288, 5.8%) (Figure 1).

Of the 684 azithromycin prescriptions, 486 (71.1%) were given *via* the intravenous route and 198 (28.9%) *via* the oral route.

### Antibiotic Prescription Pattern Based on the ATC Classification

**Figure 2:**
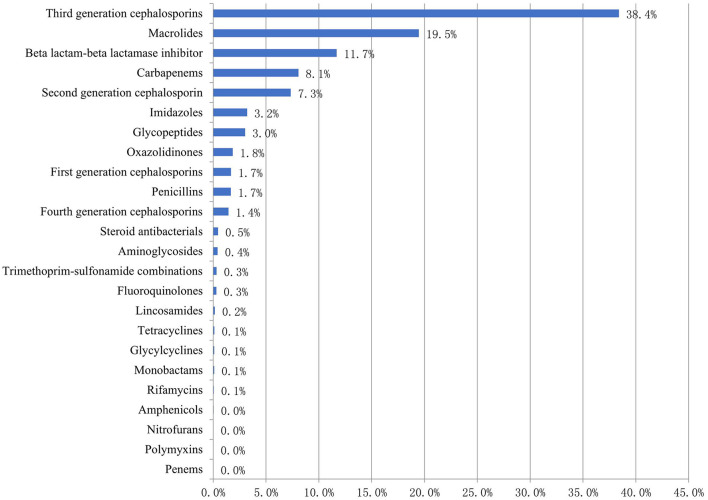
Proportion of prescribed antibiotics (ATC classification) among Chinese children in 2017–2019 (%).

Of the 4,982 antibiotic prescriptions for children, the top five classes of antibacterial agents prescribed for children were third-generation cephalosporins (1,913, 38.4%), macrolides (970, 19.5%), beta lactam-beta lactamase inhibitors (582, 11.7%), carbapenems (402, 8.1%), and second-generation cephalosporins (366, 7.3%) (Figure 2).

Of the 402 carbapenems prescriptions, meropenem was the most common, accounting for 71.6% of all carbapenems prescriptions. Of the 1,913 third generation cephalosporins prescriptions, ceftriaxone (508, 26.6%) was the most common, followed by latamoxef (403, 21.1%), cefoperazone sulbactam (335, 17.5%), ceftazidime (192, 10.0%), and ceftizoxime (185, 9.7%) (Table 3).

**Table 3:** Patterns of 4,982 antibiotic prescriptions for Chinese children by drug utilization 90% according to the ATC classification.

**ATC classes**	**Antibiotic agents**	**Frequency**	**Percentage (%)**
**Third-generation cephalosporins (*****n*** **=** **1,913)**			
	Ceftriaxone	508	26.6
	Latamoxef	403	21.1
	Cefoperazone/sulbactam	335	17.5
	Ceftazidime	192	10.0
	Ceftizoxime	185	9.7
	Cefmenoxime	166	8.7
	Cefotaxime	70	3.7
	Cefoperazone	21	1.1
	Cefixime	16	0.8
	Cefodizime	9	0.5
	Cefoperazone/tazobactam	5	0.3
	Cefdinir	2	0.1
	Cefpodoxime proxetil	1	0.1
**Macrolides (*****n*** **=** **970)**		970	
	Azithromycin	684	70.5
	Erythromycin	275	28.4
	Clarithromycin	4	0.4
	Erythromycin estolate	4	0.4
	Roxithromycin	2	0.2
	Erythromycin cyclic	1	0.1
**Beta lactam-beta lactamase inhibitor (*****n*** **=** **582)**		582	
	Amoxicillin/clavulanic acid	270	46.4
	Piperacillin/tazobactam	191	32.8
	Mezlocillin/sulbactam	78	13.4
	Amoxicillin/sulbactam	30	5.2
	Ampicillin/sulbactam	12	2.1
	Ticarcillin/enzyme inhibitor	1	0.2
**Carbapenems (*****n*** **=** **402)**			
	Meropenem	288	71.6
	Imipenem/cilastin	97	24.1
	Ertapenem	16	4.0
	Biapenem	1	0.2
**Second-generation cephalosporin (*****n*** **=** **366)**			
	Cefotiam	149	40.7
	Cefuroxime	74	20.2
	Cefminox	48	13.1
	Cefoxitin	37	10.1
	Cefmetazole	34	9.3
	Cefamandole	13	3.6
	Cefaclor	11	3.0
**Imidazoles (*****n*** **=** **160)**			
	Metronidazole	109	68.1
	Ornidazole	47	29.4
	Levoornidazole	4	2.5
**Glycopeptides (*****n*** **=** **151)**			
	Vancomycin (IV)	139	92.1
	Teicoplanin	11	7.3
	Norvancomycin	1	0.7

### Antibiotic Classes Prescribed Patterns Based on the Access/Watch/Reserve (AWaRe) Group

**Figure 3:**
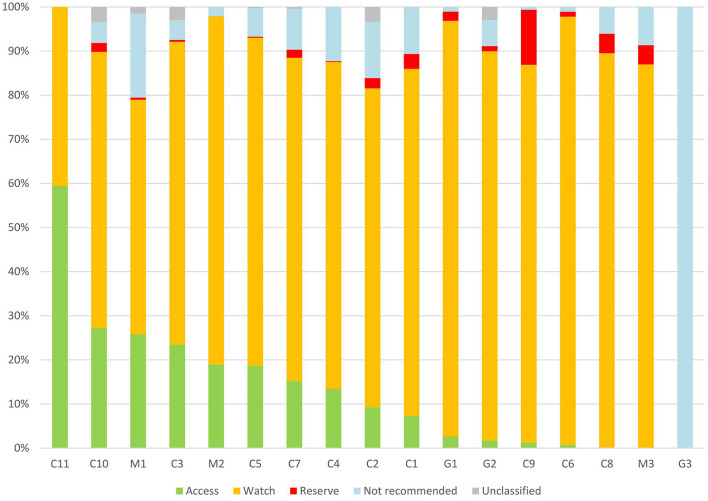
Patterns of Access, Watch, Reserve antibiotics prescribed for children from different hospitals in China. C, children's hospital; M, maternal and children's; and G, general hospital.

According to the AWaRe classification, 76 antibiotic agents were included in our survey. There were 18 (23.7%) antibiotic agents in the Access group (accounting for 12.1% of antibiotic prescriptions), 42 (55.3%) antibiotic agents in the Watch group (accounting for 76.3% of antibiotic prescriptions), and 6 (7.9%) antibiotic agents in the Reserve group (accounting for 2.1% of antibiotic prescriptions). There were three (3.9%) antibiotic agents in the not-recommended group, accounting for 8.3% of antibiotic prescriptions. 29 (38.2%) antibiotic agents of all antibiotic types accounted for 90% of all antibiotic prescriptions. The median proportion of antibiotics in the Watch group was 74.4% (95%CI, 59.6–83.7%) in different hospitals (Figure 3). The proportion of the Watch group antibiotics in general hospitals, children's hospitals, and maternal and children's hospitals was 90.4%, 76.0%, and 63.6% respectively (*P* < 0.05).

In the Access group, the commonly prescribed antibiotic agents were amoxicillin-clavulanic acid (270, 44.7%), metronidazole (109, 18.0%), and cefazolin (71, 11.8%). In the Watch group, the top-three antibiotics were azithromycin (684, 18.0%), ceftriaxone (508, 13.4%), and latamoxef (403, 10.6%). Detailed information on the antibiotic types in each group based on the WHO AWaRe classifications is shown in Table 4.

**Table 4:** 4982 antibiotics prescriptions pattern for Chinese children based on WHO AWaRe classification by drug utilization 90% in 2017–2019.

**WHO AWaRe classification**	**Antibiotic agents**	**Frequency**	**Percentage (%)**
**Access (*****n*** **=** **604)**			
	Amoxicillin/clavulanic acid	270	44.7
	Metronidazole	109	18.0
	Cefazolin	71	11.8
	Amoxicillin/sulbactam	30	5.0
	Amikacin	20	3.3
	Penicillin	17	2.8
	Amoxicillin	16	2.6
	Sulfamethoxazole/trimethoprim	16	2.6
**Watch (*****n*****=** **3,799)**			
	Azithromycin	684	18.0
	Ceftriaxone	508	13.4
	Latamoxef	403	10.6
	Meropenem	288	7.6
	Erythromycin	275	7.2
	Ceftazidime	192	5.1
	Piperacillin/tazobactam	191	5.0
	Ceftizoxime	185	4.9
	Cefmenoxime	166	4.4
	Cefotiam	149	3.9
	Vancomycin	139	3.7
	Imipenem/cilastin	97	2.6
	Cefuroxime	74	1.9
	Cefepime	72	1.9
**Reserve (*****n*** **=** **105)**			
	Linezolid	92	87.6
	Aztreonam	5	4.8
**Not recommended (*****n*** **=** **414)**			
	Cefoperazone/sulbactam	335	80.9
	Mezlocillin/sulbactam	78	18.8
**Unclassified (*****n*** **=** **60)**			
	Ornidazole	47	78.3
	Cefoperazone/tazobactam	5	8.3
	Levoornidazole	4	6.7

### Antibiotic Classes Prescribed Pattern Based on the Management of Antibiotic Classification in China

**Figure 4:**
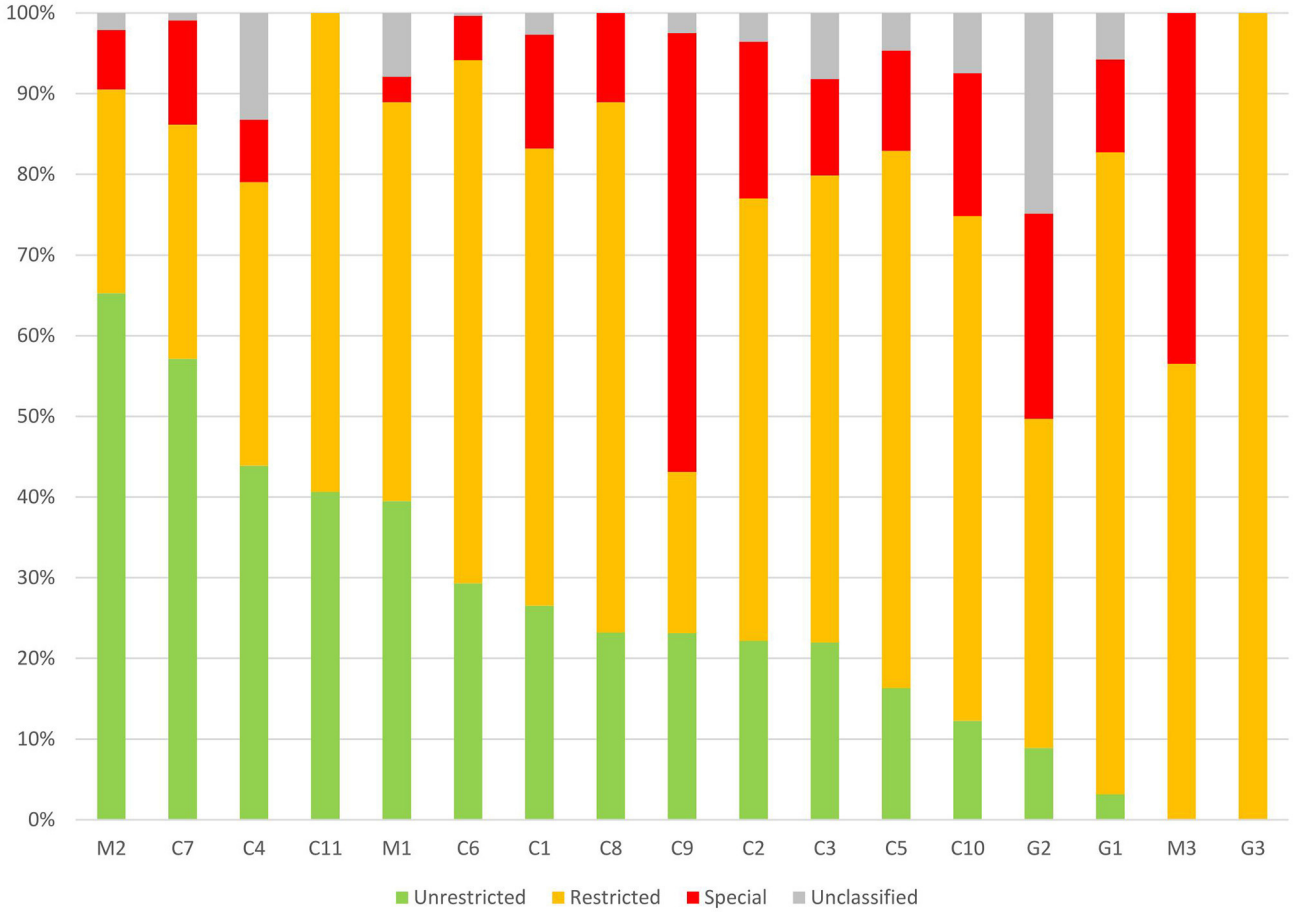
Patterns of Unrestricted, Restricted, Special antibiotics prescribed for children from different hospitals in China. C, children's hospital; M, maternal and children's; and G, general hospital.

Seventy-seven antibiotic agents were included in our survey from 2017 to 2019, and 34 (44.2%) antibiotic agents covered 90% of prescriptions. There were 15 (19.5%) types of antibiotic agents in the Unrestricted group (accounting for 26.5% of antibiotic prescriptions), 23 (29.9%) types of antibiotic agents in the Restricted group (accounting for 53.6% of antibiotic prescriptions), and 9 (11.7%) types of antibiotic agents in the Special group (accounting for 14.5% of antibiotic prescriptions). The median proportion of antibiotics in the Restricted group and Special group was 56.7% (95%CI, 44.0–64.7%) and 11.9% (95%CI, 7.8–22.6%), respectively, in different hospitals (Figure 4). The proportion of the Special group antibiotics in general hospitals, children's hospitals, and maternal and children's hospitals was 17.9, 14.7, and 7.5% (*P* < 0.05).

In the Unrestricted group (*n* = 1,319), the commonly prescribed antibiotic agents were ceftriaxone (508, 38.5%), erythromycin (275, 20.9%), and azithromycin (for oral use 198, 15.0%). In the Restricted group, the top-three antibiotics were azithromycin (for intravenous injection 486, 18.2%), latamoxef (403, 15.1%) and cefoperazone-sulbactam (335, 12.5%). In the Special group, the top-three antibiotics were meropenem (288, 39.9%), vancomycin (139, 19.3%) and imipenem-cilastatin (97, 13.5%).

Detailed information on the antibiotic types in each group based on the antibiotic classification in China is shown in Table 5.

**Table 5:** 4,982 antibiotics (China classification) prescribed to Chinese children by drug utilization 90% in 2017–2019.

**China classification**	**Antibiotic agents**	**Frequency**	**Percentage (%)**
**Unrestricted (*****n*** **=** **1,319)**			
	Ceftriaxone	508	38.5
	Erythromycin	275	20.8
	Azithromycin(oral)	198	15.0
	Metronidazole	109	8.3
	Cefuroxime	74	5.6
	Cefazolin	71	5.4
**Restricted (*****n*** **=** **2,670)**			
	Azithromycin(intravenous)	486	18.2
	Latamoxef	403	15.1
	Cefoperazone/sulbactam	335	12.5
	Amoxicillin/clavulanic acid	270	10.1
	Ceftazidime	192	7.2
	Piperacillin/tazobactam	191	7.2
	Ceftizoxime	185	6.9
	Cefmenoxime	166	6.2
	Cefotiam	149	5.6
	Mezlocillin/sulbactam	78	2.9
**Special (*****n*** **=** **721)**			
	Meropenem	288	39.9
	Vancomycin	139	19.3
	Imipenem-cilastin	97	13.5
	Linezolid	92	12.8
	Cefepime	72	10.0
**Unclassified (*****n*** **=** **272)**			
	Cefminox	48	17.6
	Ornidazole	47	17.3
	Cefoxitin	37	13.6
	Cefmetazole	34	12.5
	Amikacin	20	7.4
	Cefamandole	13	4.8
	Levofloxacin	12	4.4
	Sulbenicillin	9	3.3
	Flucloxacillin	7	2.6
	Azlocillin	5	1.8
	Aztreonam	5	1.8
	Doxycycline	5	1.8
	Cefoperazone/tazobactam	5	1.8

### The Antibiotic Prescriptions Pattern for Acute Pneumonia in Chinese Children

A total of 2,632 antibiotic prescriptions (accounting for 52.8% of the total prescriptions in our study) were for acute pneumonia in Chinese children. Pneumonia was the most common indication for antibiotics in Chinese children. A total of 2,632 antibiotic prescriptions were for 2,142 children suffering from pneumonia. A total of 434 (16.5%) antibiotic prescriptions in 326 (15.2%) children were a targeted application and 2,198 (83.5%) antibiotic prescriptions in 1,816 (84.8%) children were based on empirical use. The five most frequently prescribed antibiotic agents were azithromycin (566, 21.5%), ceftriaxone (282, 10.7%), latamoxef (228, 8.7%), amoxicillin-clavulanic acid (205, 7.8%), and erythromycin (202, 7.7%). The top-five antibiotic classes prescribed for children were third-generation cephalosporins (996, 37.8%), macrolides (777, 29.5%), beta lactam-beta lactamase inhibitors (368, 14.0%), carbapenems (165, 6.3%) and second-generation cephalosporins (95, 3.6%).

Of the 2,632 antibiotic prescriptions for pneumonia in Chinese children, 13 antibiotic agents included in the Access group accounted for 10.6%, whereas 35 antibiotic agents in the Watch group accounted for 78.4% of all antibiotic prescriptions. In the Watch group, azithromycin (566, 27.4%) was the most commonly prescribed antibiotic, followed by ceftriaxone (282, 13.7%), and latamoxef (228, 11.1%).

Of the 2,632 antibiotic prescriptions for pneumonia, 14 antibiotic agents included in the Unrestricted group accounted for 26.9%, whereas 20 antibiotic agents included in the Restricted group accounted for 58.9%. The Special group antibiotics (which were restricted and authorized only by consultants) accounted for 11.8%.

## Discussion

In December 2,011, the National Health Commission of the People's Republic of China initiated a special campaign to restrict the use of antibiotics. The antibiotic utilization rate and antibiotic use density for children and adults showed an apparent decline in some hospitals (4, 5).

However, the proportion of antibiotic-resistance bacteria producing extended-spectrum beta-lactamases and carbapenem-resistant organisms remains high and has been increasing year by year in children. The resistance of *Escherichia coli* to ceftriaxone and meropenem was 52.3–56.9% and 2.4–4.3% isolated in Chinese children from 2017 to 2019. The resistance of *Klebsiella pneumoniae* to ceftriaxone and meropenem has been documented in 56.1–60.9% and 19.8-23.6% of isolated Chinese children from 2017 to 2019 (6–8).

Antimicrobial resistance has a relationship with antibiotics exposure, but also to classes of antibiotics. Therefore, antibiotics with a high potential for antimicrobial resistance are likely to be overused, which promotes the production of antimicrobial-resistant pathogens.

In general, Watch group antibiotics are broad-spectrum with higher potential for antimicrobial resistance and their use should be restricted. Of the all antibiotic prescriptions for Chinese children in our survey, the proportion of Watch-group antibiotics with high potential for antimicrobial resistance potential was 76.3%, whereas Access-group antibiotics accounted for only 12.12%. Usually, Access-group antibiotics are recommended as first-line antibiotics for children with common infectious diseases, and the WHO has recommended that 60% of all antibiotic consumption must come from the Access group antibiotics (9). This target was not entirely applicable to this survey, because the antibiotic prescriptions were only for hospitalized children. The data from a global survey showed the proportion of the Access group antibiotics for Chinese children was very low. In a worldwide point-prevalence survey on antibiotic prescriptions for hospitalized children and neonates in 2016, the proportion of Access antibiotic use in China (7.8%) was the lowest and the Watch group antibiotics in China (74.1%) was the second-highest among the observed countries (10).

The main reason for the observation stated above was the low use of penicillin and aminoglycosides, which are included in the Access group for children in China. Conversely, more third-generation cephalosporins and macrolides were prescribed for children in China. A skin test before penicillin is prescribed may be the main barrier. A skin test must be taken before the use of penicillin (i.v., or p.o.) in China, but this is not required in other countries (11). The inconvenience of using penicillin and false-positive skin tests could lead physicians and patients' parents to choose cephalosporins or macrolides. Some studies have recommended that the penicillin skin test should be used for patients with a history of penicillin allergy, not for all children (12).

In our survey, the proportion of aminoglycosides was only 0.42% (21/4,982). Aminoglycosides were one of the most commonly prescribed antibiotic classes in countries such as India (10.4%) and Australia (14%) (13, 14). Virtual non-use of aminoglycosides in China may have been led by China's antimicrobial-management policies for children. Gentamicin is prohibited in children under 6 years of age in China because of probable ototoxicity (15), but there was no evidence supporting this policy. The ototoxicity of aminoglycosides such as gentamicin is associated with susceptibility to the gene *A1555G*. Compared to European children (0.19%), the carrier rate of *A1555G* in Chinese newborns (0.12%) was lower (16, 17). According to a microbiological survey of Chinese children, *E.coli*. was the most common pathogen, accounting for 12.5% of all isolates, 38.4% of isolates from urine, and 35.5% from pus. The resistance of *E.coli*. to ceftriaxone, amikacin, and gentamicin was 52.3, 1.3, and 34.9%, respectively. The resistance of *K. pneumoniae* to ceftriaxone, amikacin, and meropenem was 56.1, 5.9, and 20.6%, respectively. Hence, amikacin was an option, especially for carbapenem-resistant *K. pneumoniae*.

It was likely that the third-generation cephalosporins included in the Watch group in Chinese children were overused. In our survey, third-generation cephalosporins accounted for 38.4% (1913/4982) of all antibiotic prescriptions. The proportion of third-generation cephalosporins prescribed to children in China in our survey is higher than that in Australia (10.20%), Ghana (18.5%), and Italy (26.3%) (13, 18, 19). Extensive use of third-generation cephalosporins would cause an increase in antibiotic-resistant bacteria. In a surveillance study from 10 children's hospitals, the resistance proffered by *E.coli*. and *K. pneumoniae* to ceftriaxone was >50% (8).

In this survey, the children with pneumonia were the main population prescribed antibiotics. The most frequently prescribed antibiotic class for pneumonia was third-generation cephalosporins (37.8%). Ampicillin-sulbactam was the most frequently prescribed antibiotic for children with pneumonia in Japan (20). According to a report from the National Antimicrobial Resistance Monitoring Network, for Chinese children in 2019, the most common bacteria isolated from sputum specimens in children were *Streptococcus pneumoniae* (21.2%), *Haemophilus influenza* (21.1%), and *Staphylococcus aureus* (11.9%), and the proportions of penicillin-resistant and penicillin-intermediate to *Streptococcus pneumoniae* which was the most common bacteria for pneumonia in children were 15.9% and 1.2% (8). Amoxicillin-clavulanic acid (an Access-group antibiotic) would be efficacious in most isolates from Chinese children suffering from pneumonia. Moreover, some infections caused by penicillin-intermediate *Streptococcus pneumoniae* will be effective by increasing the dose of amoxicillin. The antibiotics prescribed were not adapted to the pattern of antimicrobial resistance in Chinese children. In the future, there are some ways to decrease the use of third-generation cephalosporins and increase the proportion of penicillins. For pneumonia in children, clinical characteristics and antimicrobial resistance of pathogens isolated from sputum should be combined as the evidence to prescribe antibiotics. Penicillins with or without enzyme inhibitors should be the first-line treatment or primary choice for pneumonia. In addition, the antibiotics used widely for Chinese children, such as latamoxef (8.1%), were rarely used in North America or Europe, possibly because of a higher incidence of side effects, such as bleeding (10). The antibiotics listed in the essential medicines should be selected and side effects monitored closely.

Macrolides are Watch group antibiotics. They accounted for 19.5% of antibiotics and were the second most commonly prescribed antibiotic class in our survey. Azithromycin accounted for 13.7% of all single prescriptions of antibiotics. The proportion of azithromycin was far higher than that in Africa (2.1%), North America (3.2%), the Eastern Mediterranean (3.4%), Europe (3.4%), and South-East Asia (1.9%) (10). Macrolides (especially azithromycin) may be overused in Chinese children. Usually, macrolides are prescribed to children with a respiratory tract infection caused by *Mycoplasma pneumonia* and a positive penicillin skin test. Azithromycin has been misused because of misinterpretation of test results for *Mycoplasma pneumoniae*. Many physicians incorrectly take the positivity of IgG or IgM to *Mycoplasma pneumoniae* as the only indication for prescribing macrolides. We showed that >70% of azithromycin prescriptions were for intravenous use. Intravenous azithromycin is off-label and can cause adverse cardiovascular effects in children (21). The appropriateness of macrolide prescribing should be analyzed to reduce overuse. This can be achieved through case series and cohort studies, which can be used to analyze the indications for macrolides prescription and the course of treatment.

According to the Management of Antibiotic Classification in China, 14.5% of antibiotic prescriptions belonged to the Special group antibiotics. They are expensive, have a higher probability of inducing bacterial resistance, and their use is restricted in China. Of the antibiotic prescriptions for common and non-invasive infections, prescriptions for Special-group antibiotics accounted for 11.8%. In the Special group, meropenem (5.8%) was the most commonly prescribed antibiotic, followed by vancomycin (2.8%). The proportion of prescriptions for meropenem and vancomycin of all prescriptions was higher than that for meropenem (4.5%) and vancomycin (2.0%) in India, which has a high prevalence of antimicrobial resistance (14). It was likely that carbapenems are overused, thereby leading to a rapidly rising prevalence of pathogens resistant to carbapenems. The overall prevalence of carbapenem-resistant *Enterobacterales* increased from 3.0% in 2005 to 10.2% in 2017 (22). The proportions of carbapenem-resistant Klebsiella pneumoniae (CRKP) and Acinetobacter baumannii (CRAB) showed decreasing trends were observed (23). But the average proportion of CRKP and CRAB were still very high (19.3% and 45.4%, respectively). The Special group antibiotic prescriptions such as carbapenems and vancomycin should be evaluated by an antimicrobial stewardship organization in hospitals to decrease usage.

We found that 28.3% of antibiotic prescriptions were prescribed for hospital-acquired infections (HAIs) in children, data from the worldwide antibiotic resistance and prescribing in European children (ARPEC) (24), while 9.1% of antibiotic prescriptions were for children with HAIs in India, from the global antibiotic resistance and prescribing in children and neonates (GARPEC) data (14). In this survey, only 1.7% of antibiotic prescriptions were for HAIs in Chinese children. The probable reason was that patients with long hospital stays and a high risk of nosocomial infection admitted to the pediatric hematology and oncology wards were not included in our survey. In future research, all patients in different specialties should be included to decrease the bias.

In this study, 76 antibiotic types were prescribed in different hospitals. It seems probable that too many types of antibiotics were prescribed for Chinese children. Of course, not all of the types were used in a single hospital, but our survey showed that antibiotics prescribed in different regions and hospitals varied greatly. A national essential medicine list of antibiotics for children should be formulated and extended to all hospitals. In addition, 30 antibiotics belonging to the unclassified group accounted for 5.46% of total antibiotic prescriptions according to China's classification. This meant many kinds of antibiotics were not in the classified groups and accounted for a small percentage. Some antibiotic agents are not classified into groups, because they are not commonly used for children like aminoglycosides and quinolones in China. This was not beneficial for antibiotic stewardship. In fact, all antibiotics should be classified into groups for encouraging optimal use and decreasing the unnecessary kinds of antibiotics prescribed by standardized training for doctors.

The WHO AWaRe classification system ranks antibiotics to enable the pattern of antibiotic prescribing to be seen clearly and to facilitate the creation of an antimicrobial strategy. However, carbapenems and glycopeptides (which should be a last resort of alternative drugs for children with multi-drug resistance pathogens) are put in the Watch group together with third-generation cephalosporins and macrolides. Making the difference clear based on the AWaRe classification is difficult. China's antibiotics management system classifies this antibiotic group separately as special use grade. A combination of the two classification systems could display the distribution of broad-spectrum antimicrobial agents readily, and help to formulate targeted management strategies.

This study had several strengths. First, we focused on the distribution of antibacterial agents for Chinese children hospitalized in tertiary hospitals. This design reduced the bias of surveillance of data from different-level hospitals and different populations. Second, a standardized and structured point-prevalence survey was applied to collect data from 17 hospitals located in 10 provinces in China. This approach contributed to the reliability of data, a large dataset, and the representativeness of antibiotic prescriptions for children in China. Third, this is the first study to describe the prescription pattern of different types of antibiotic agents for children by combining the ATC classification, WHO AWaRe, and the Management of Antibiotic Classification in China.

The present study had several limitations. First, a lack of microbiology data, antimicrobial susceptibility results, and detailed patient characteristics hampered determination of the appropriateness of antibiotic use. Second, this was a cross-sectional survey, so data on the duration of antibiotic therapy and switching patterns were not collected. Third, the participating hospitals contributed the data voluntarily, and the eligibility of such data might not have been uploaded on the server. Fourth, the majority of hospitals joined in this survey located in northern, eastern, and southern China while only two hospitals were from western China. Thus, the results might not be representative of all of China. This study did not include all inpatient wards like hematology and oncology wards. In the next surveys, more details on antibiotic prescribing such as duration of treatment, pathogens, and underlying diseases will be collected and more hospitals, especially those located in western China and rural districts, will be added to the monitoring network to increase the representativeness.

We provided baseline data on antibiotic prescriptions in the real world. Targeted antimicrobial stewardship can be formulated using our data. The aims of the strategies are to reduce the use of broad-spectrum antibiotic agents and increase the use of narrow-spectrum antibiotics to avoid further exacerbations of antimicrobial resistance. At national and regional levels, physicians, pharmacologists, microbiologists and policymakers, and others should collaborate to improve the quality of antibiotic prescribing for Chinese children.

## Data Availability Statement

The raw data supporting the conclusions of this article will be made available by the authors, without undue reservation.

## Ethics Statement

The studies involving human participants were reviewed and approved by the Ethics Committees at Shenzhen Children's Hospital (201801504). Written informed consent to participate in this study was provided by the participants' legal guardian/next of kin.

## Author Contributions

YY, JZ, WZ, YZ, and JD were responsible for the study concept and design. YY organized all hospitals to collect data. JZ, XM, LT, DT, LL, YL, GL, LS, WZ, JQ, GL, WL, QC, and LW collected the data of antibiotic prescriptions and edited the manuscript. JZ and KW contributed to the data management and analyses. JZ contributed to the interpretation of data and writing of the manuscript. YY, YZ, and JD revised the manuscript. All authors reviewed and agreed the final manuscript.

## Funding

This research was funded by Project of the Expert Committee on Clinical Application and Drug Resistance Evaluation of Antimicrobial Drugs of the National Health Commission (No. KJYWZWH-OT-02-2021-01), Shenzhen Healthcare Research Project (No. SZLY2018016), and Shenzhen Fund for Guangdong Provincial High level Clinical Key Specialties (No. SZGSP012). The sponsors had no any role in the study design, data collection, data analysis, data interpretation, writing of report, or decision to submit the article for publication.

## Conflict of Interest

The authors declare that the research was conducted in the absence of any commercial or financial relationships that could be construed as a potential conflict of interest.

## Publisher's Note

All claims expressed in this article are solely those of the authors and do not necessarily represent those of their affiliated organizations, or those of the publisher, the editors and the reviewers. Any product that may be evaluated in this article, or claim that may be made by its manufacturer, is not guaranteed or endorsed by the publisher.
