# Supplementary material for: Pattern of Antibiotic Prescriptions in Chinese Children, A Cross-Sectional Survey From 17 Hospitals Located Across 10 Provinces of China
Source: Front Pediatr. 2022 Jul 14;10:857945. doi: 10.3389/fped.2022.857945 (PMC10155817; doi:10.3389/fped.2022.857945)
Supplement: Supplementary file 1 [file Table_1.DOCX]

Supplementary table 1 A list of antimicrobial agents prescribed in this study based on WHO AWaRe classification and Management of Antibiotic Classification in China

| Antibiotic | AWaRe | Category in China |
| --- | --- | --- |
| Amikacin | Access | Unclassified |
| Amoxicillin | Access | Unrestricted |
| Amoxicillin/clavulanic Acid | Access | Restricted |
| Amoxicillin/sulbactam | Access | Restricted |
| Ampicillin | Access | Unrestricted |
| Ampicillin-sulbactam | Access | Restricted |
| Azithromycin(Intravenous) | Watch | Unrestricted |
| Azithromycin(Oral) | Watch | Restricted |
| Azlocillin | Watch | Unclassified |
| Aztreonam | Reserve | Unclassified |
| Biapenem | Watch | Unclassified |
| Cefaclor | Watch | Unrestricted |
| Cefamandole | Watch | Unclassified |
| Cefathiamidine | Access | Restricted |
| Cefazolin | Access | Unrestricted |
| Cefdinir | Watch | Restricted |
| Cefepime | Watch | Special |
| Cefixime | Watch | Restricted |
| Cefmenoxime | Watch | Restricted |
| Cefmetazole | Watch | Unclassified |
| Cefminox | Watch | Unclassified |
| Cefodizime | Watch | Restricted |
| Cefoperazone | Watch | Restricted |
| Cefoperazone-sulbactam | Not recommended | Restricted |
| Cefoperazone-tazobactam | Unclassified | Unclassified |
| Cefotaxime | Watch | Restricted |
| Cefotiam | Watch | Restricted |
| Cefoxitin | Watch | Unclassified |
| Cefpodoxime proxetil | Watch | Restricted |
| Ceftazidime | Watch | Restricted |
| Ceftezole | Access | Unclassified |
| Ceftizoxime | Watch | Restricted |
| Ceftriaxone | Watch | Unrestricted |
| Cefuroxime | Watch | Unrestricted |
| Chloramphenicol | Access | Restricted |
| Ciprofloxacin | Watch | Unclassified |
| Clarithromycin | Watch | Unrestricted |
| Clindamycin | Access | Unrestricted |
| Cloxacillin | Access | Unclassified |
| Doxycycline | Access | Unclassified |
| Ertapenem | Watch | Special |
| Erythromycin | Watch | Unrestricted |
| Erythromycin cyclic | Unclassified | Unclassified |
| Erythromycin estolate | Watch | Unrestricted |
| Faropenem | Reserve | Unclassified |
| Flucloxacillin | Access | Unclassified |
| Fluloxacillin-amoxicillin | Not recommended | Unclassified |
| Furazolidone | Unclassified | Unclassified |
| Fusidic ccid | Watch | Restricted |
| Imipenem-cilastin | Watch | Special |
| Latamoxef | Watch | Restricted |
| Levofloxacin | Watch | Unclassified |
| Levoornidazole | Unclassified | Unclassified |
| Linezolid | Reserve | Special |
| Meropenem | Watch | Special |
| Metronidazole | Access | Unrestricted |
| Mezlocillin | Watch | Restricted |
| Mezlocillin-sulbactam | Not recommended | Restricted |
| Minocycline | Reserve | Unclassified |
| Moxifloxacin | Watch | Unclassified |
| Norvancomycin | Unclassified | Special |
| Ornidazole | Unclassified | Unclassified |
| Oxacillin | Access | Unclassified |
| Penicillin | Access | Unrestricted |
| Piperacillin | Watch | Restricted |
| Piperacillin-tazobactam | Watch | Restricted |
| Polymyxin B | Reserve | Unclassified |
| Rifamycin | Watch | Unclassified |
| Rifaximin | Watch | Unclassified |
| Roxithromycin | Watch | Unrestricted |
| Sulbenicillin | Watch | Unclassified |
| Teicoplanin | Watch | Special |
| Ticarcillin and enzyme inhibitor | Unclassified | Unclassified |
| Tigecycline | Reserve | Special |
| Tobramycin | Watch | Unclassified |
| Trimethoprim-sulfamethoxazole | Access | Unrestricted |
| Vancomycin | Watch | Special |
